# A Novel *Zak* Knockout Mouse with a Defective Ribotoxic Stress Response

**DOI:** 10.3390/toxins8090259

**Published:** 2016-09-02

**Authors:** Dakshina M. Jandhyala, John Wong, Nicholas J. Mantis, Bruce E. Magun, John M. Leong, Cheleste M. Thorpe

**Affiliations:** 1 Department of Molecular Biology and Microbiology, Tufts University School of Medicine, Boston, MA 02111, USA; john.leong@tufts.edu; 2 Division of Geographic Medicine and Infectious Diseases, Tufts Medical Center, Boston, MA 02111, USA; cthorpe@tuftsmedicalcenter.org; 3 School of Nursing, MGH Institute of Health Professions, Boston, MA 02129, USA; jwong1@mghihp.edu (J.W.); bmagun@mghihp.edu (B.E.M.); 4 Division of Infectious Disease, Wadsworth Center, New York State Department of Health, Albany, NY 12208, USA; nicholas.mantis@health.ny.gov

**Keywords:** ZAK, MAP3K, Ricin, Ribotoxic Stress Response, protein synthesis inhibition, p38, JNK1/2, inflammation, murine, macrophage

## Abstract

Ricin activates the proinflammatory ribotoxic stress response through the mitogen activated protein 3 kinase (MAP3K) ZAK, resulting in activation of mitogen activated protein kinases (MAPKs) p38 and JNK1/2. We had a novel *zak−/−* mouse generated to study the role of ZAK signaling in vivo during ricin intoxication. To characterize this murine strain, we intoxicated *zak−/−* and *zak+/+* bone marrow–derived murine macrophages with ricin, measured p38 and JNK1/2 activation by Western blot, and measured *zak*, *c-jun*, and *cxcl-1* expression by qRT-PCR. To determine whether *zak−/−* mice differed from wild-type mice in their in vivo response to ricin, we performed oral ricin intoxication experiments with *zak+/+* and *zak−/−* mice, using blinded histopathology scoring of duodenal tissue sections to determine differences in tissue damage. Unlike macrophages derived from *zak+/+* mice, those derived from the novel *zak−/−* strain fail to activate p38 and JNK1/2 and have decreased *c-jun* and *cxcl-1* expression following ricin intoxication. Furthermore, compared with *zak+/+* mice, *zak−/−* mice have decreased duodenal damage following in vivo ricin challenge. *zak−/−* mice demonstrate a distinct ribotoxic stress–associated phenotype in response to ricin and therefore provide a new animal model for in vivo studies of ZAK signaling.

## 1. Introduction

Ricin is a plant lectin extracted from the beans of *Ricinis communis*, a common ornamental cultivated by horticulture enthusiasts and the industrial source of castor oil. Ricin is a select agent toxin listed as a Category B potential bioterrorism agent by the US Center for Disease Control and Prevention. Historically there have been several instances of use or intended use of ricin as a weapon [1], and within the last 15 years, there have been several news reports concerning the use or possession of ricin as a bioterror agent [2,3,4,5,6,7,8]. Despite its potential to inflict mass casualties during a terrorist attack, current treatment options for ricin intoxication are limited to supportive care. 

Ricin damages cells through N-glycosidase–mediated depurination of a single adenine from the alpha-sarcin/ricin loop of the 28S ribosomal RNA subunit, leading to stalling of actively translating ribosomes and inhibition of protein synthesis [9,10,11]. The damage to actively translating ribosomes has been shown to activate the ribotoxic stress response (RSR), defined by the activation of one or more of the mitogen activated protein kinases (MAPKs) following intoxication by a subset of protein synthesis inhibitors [9,12,13]. Protein synthesis inhibition, particularly that resulting from ribosomal insult, is a common strategy employed by various toxins, antibiotics, viruses, and bacterial effectors, so the host’s ability to detect and respond to ribosomal insult may represent a unique innate immune response. The RSR is associated with induction of pro-apoptotic signaling and activation of a pro-inflammatory response, including the production of pro-inflammatory cytokines [14,15,16,17,18,19,20,21,22]. The induction of pro-inflammatory cytokines may be perceived as paradoxical given the inhibition of global translation by RSR-inducing agents such as ricin.

Inflammatory responses have been associated with both oral and pulmonary ricin intoxication models [23,24]. Oral administration of ricin was shown to be associated with increased duodenal macrophage chemotactic protein 1 (MCP-1) [23]. In a pulmonary model, ricin intoxication resulted in the increased expression levels of inflammatory cytokines in the lung, infiltration of neutrophils in the lung parenchyma and bronchoalveolar lavage fluid, and elevated extravascular protein leakage across the lung endothelial barrier; however, depletion of macrophages prior to ricin intoxication was associated with a reduction of inflammatory transcripts, decreased neutrophilia, and decreased microvascular permeability [24]. Therefore, the RSR may contribute to ricin-associated disease in part by promoting inflammation.

It has been demonstrated in intestinal epithelial cells and Vero cells that the ricin-induced RSR is mediated by the mitogen activated protein 3 kinase (MAP3K) ZAK, also termed MRK, MLK7, AZK, or MLTK [25,26,27,28], and that inhibition of ZAK signaling in intestinal epithelial cells perturbs RSR-associated IL-8 gene transcription and protein production, and pro-apoptotic signaling [29]. In this study, we evaluated the in vitro and in vivo responses of a novel *zak−/−* murine strain to ricin intoxication.

## 2. Results

### 2.1. Bone Marrow–Derived Macrophages (BMDMs) from zak−/− Mice Have a Distinct Defect in JNK1/2 and p38 Activation and Associated Gene Expression Following Ricin Intoxication

Because it was previously demonstrated in vitro, using the transformed cell line HCT-8, that ZAK was a key intermediate of ricin-induced MAPK activation with subsequent IL-8 gene expression and protein production [29], we had the Texas Institute for Genomic Medicine (TIGM) generate a *zak+/−* mouse, so we would have a genetic model with which to study the role of ZAK in vivo. Upon receiving mating pairs of *zak+/−* mice of a mixed 129S5 and C57BL/6 genetic background (129/B6), the mice were crossed to generate a population of *zak−/−* mice. Pilot experiments with BMDMs from *zak−/−* mice of the mixed 129/B6 genetic background or noncongenic wild-type C57BL/6 (B6) mice demonstrated that *zak−/−* BMDMs had a defective RSR (Figure S1), as evidenced by the absence of p38 and JNK1/2 activation following intoxication with ricin. However, p38 and JNK1/2 were induced in *zak−/−* 129/B6–derived BMDMs following treatment with a variety of other pro-inflammatory stimuli including lipopolysaccharide (LPS) (Figure S1), thereby specifically implicating a defect in ZAK signaling and the RSR in the *zak−/−* strain. Furthermore, expression of several pro-inflammatory cytokine genes known to be induced by ricin or other ribotoxic stressors such as deoxynivalenol (DON) was observed to be lower in BMDMs from *zak−/−* 129/B6 mice, as compared to noncongenic wild-type B6 controls (Figure S2).

**Figure 1:**
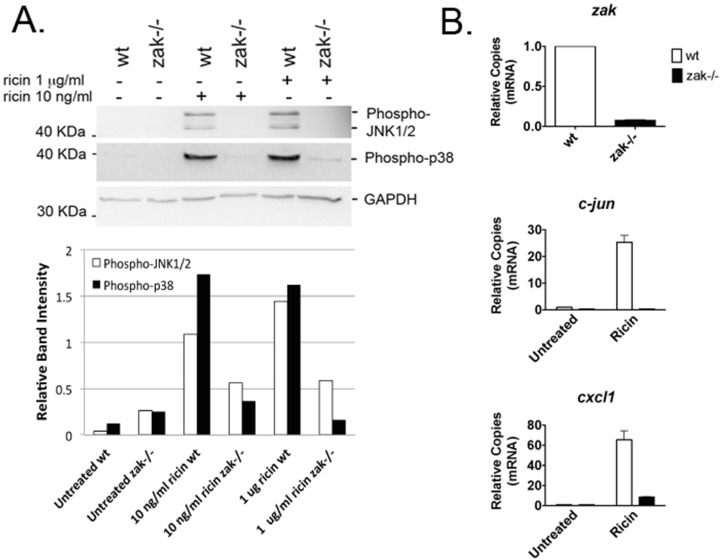
BMDMs from C57BL/6 *zak−/−* mice do not induce the RSR or RSR-associated gene expression. Panel A: Western blot for phosphorylated JNK1/2 and p38 performed following intoxication of wild-type *zak+/+* (wt) or *zak−/−* BMDMs with 1 μg/mL (~17 μM) or 10 ng/mL (~0.17 μM) ricin for 4 h in serum-free BM culture media. Band intensity for phosphorylated JNK1/2 and p38 was normalized against that for GAPDH loading controls. Normalized band intensity is shown for phosphorylated JNK1/2 (white bars) and phosphorylated p38 (black bars). Band intensity was measured using ImageJ 1.46r software (National Insitutes of Health, Bethesda, MD, USA). Panel B: qRT-PCR was used to detect *zak*, *c-jun*, and *cxcl-1* mRNAs from wild-type or *zak−/−* BMDMs. *β-actin* was used as a reference gene. Ricin intoxication was performed in BM culture media with the addition of 10 ng/mL (~0.17 μM) ricin for a period of 4 h. The white and black bars represent datum from wt and *zak−/−* BMDMs, respectively. The graphs were generated from three technical replicates for each target mRNA. For Panels 1A and 1B, numerical data were processed using Microsoft Excel for Mac, Version 14.6.5, 2011 (Microsoft Corporation, Cambridge, MA, USA) and Prism for Mac, Version 5.0d, 2010 (GraphPad Software, Inc., La Jolla, CA 92037 USA), respectively.

Based on these promising findings, we backcrossed the *zak−/−* mice over 11 generations onto a C57BL/6 genetic background, and mated the resulting heterozygote *zak+/−* mice to generate a congenic C57BL/6 *zak−/−* population. The C57BL/6 *zak−/−* strain displayed no defect in fecundity or overall growth and development. However, compared to that of wild-type C57BL/6 mice (i.e., *zak+/+*), BMDMs from the *zak−/−* C57BL/6 mice demonstrated a distinct absence of the RSR following ricin treatment, as measured by absent or decreased phosphorylation of p38 and JNK1/2 (Figure 1A). These results mirror previous studies in HCT-8 and Vero cells, where pretreatment with the ZAK-specific inhibitor DHP-2 or siRNA knockdown of *zak* blocked or decreased ricin-induced p38 and JNKs activation [29]. 

In previous studies using the ZAK inhibitor DHP-2, it was demonstrated that DHP-2 pretreatment also caused a decrease in ricin-mediated IL-8 gene expression and protein production [29]. Therefore, we decided to determine whether *zak−/−* BMDMs had perturbed expression of *c-jun* and *cxcl1*, the latter of which is a functional murine homolog of human IL-8 and a potent neutrophil chemoattractant. Phosphorylation of c-jun by JNK1/2 promotes activation of the transcription factor AP-1, of which c-jun is a component, and thereby contributes to its own up-regulation as well as that of several other genes including that of proinflammatory cytokines [30]. In addition, *c-jun* can be up-regulated downstream of p38 activation. We also measured *zak* expression as an additional verification that the gene trap insertion was blocking or down-regulating *zak* expression. Consistent with DHP-2 treatment of HCT-8 cells [29] and unlike *zak+/+* BMDMs, the deficit in *zak* expression by *zak−/−* BMDMs correlated with a large decrease in ricin-induced *c-jun* and *cxcl-1* (Figure 1B). These data suggest that cells from the *zak−/−* mouse have a ZAK-specific phenotype and that the *zak−/−* mouse is a suitable model to study the role of ZAK in ricin intoxication.

### 2.2. zak−/− Mice Demonstrate Decreased Epithelial Damage When Gavaged with Ricin

**Figure 2:**
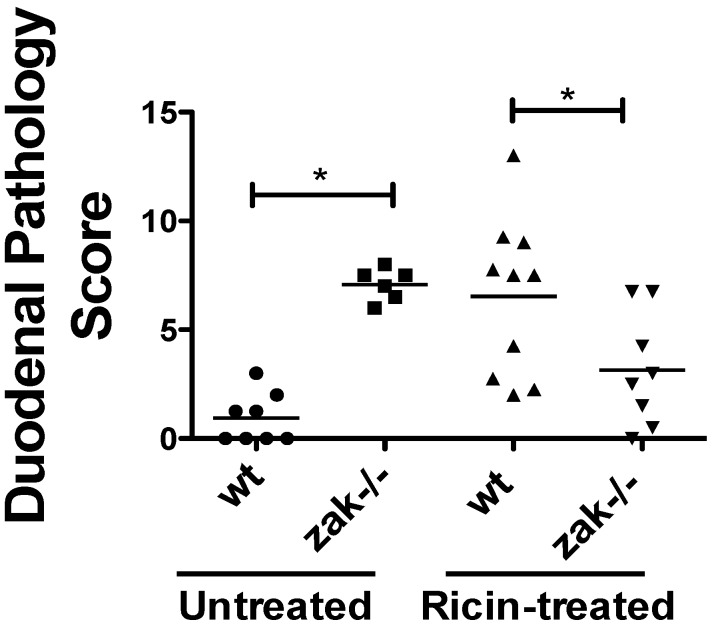
C57BL/6 backcrossed *zak−/−* mice have a lower duodenal pathology score than *zak+/+* (wt) mice following oral intoxication with ricin. Mice were gavaged with 10 mg/kg ricin (oral murine LD_50_ ~10–30 mg/kg [23,33,34]) or vehicle (PBS) and sacrificed ~22 h following gavage. Histopathology scoring as defined in Methods was performed on H&E stained duodenal sections. The number of animals in each group are as follows: wt gavaged with vehicle = 8; *zak−/−* gavaged with vehicle = 6; wt gavaged with ricin = 10; *zak−/−* gavaged with ricin = 8. Horizontal lines mark the mean score for each treatment group. Data reflect a compilation of two independent experiments. * Indicates significance *p* < 0.05 by one-way ANOVA and Tukey’s multiple comparison test. Statistical analysis was performed using Prism for Mac, Version 5.0d, 2010 (GraphPad Software, Inc., La Jolla, CA 92037 USA).

Because the ribotoxic stress response is associated with both inflammatory and apoptotic responses [12,31], we hypothesized that a deficiency in this innate response to ribosomal damage would result in reduced ricin-associated tissue damage. Therefore, we employed a mouse model of oral ricin intoxication described by Yoder et al. [23] and also by Flora et al. [32]. Following oral ricin administration we found that *zak−/−* mice did indeed have a decrease in duodenal pathology compared to congenic wild-type *zak+/+* mice (Figure 2). The data suggests that the *zak−/−* mice have a distinct ZAK-associated phenotype, possibly resulting in the observed decrease in epithelial abnormalities during ricin intoxication. Interestingly, vehicle-treated *zak−/−* mice demonstrated increased histopathologic abnormalities compared with vehicle-treated *zak+/+* mice.

## 3. Discussion

Bone marrow–derived macrophages from the *zak*-knockout mouse are incapable of mounting a ricin-mediated RSR, and *zak*-knockout mice appear to sustain less ricin-associated duodenal damage following intoxication when compared to wild-type animals. The observed mitigation in duodenal damage may occur as a result of an attenuated pro-inflammatory response resulting from the ablated RSR. Our in vitro results suggest that ricin-induced CXCL-1 may be severely perturbed in the *zak−/−* mouse. Since CXCL-1 is a potent neutrophil chemoattractant, it is tempting to speculate that *zak* deficiency results in a decreased neutrophil response to ricin-mediated cellular damage. With less neutrophil infiltration, epithelial barrier function may be better maintained resulting in less net duodenal damage. However, future studies are needed to verify that a defect in neutrophil infiltration occurs and/or contributes to the decrease in duodenal damage seen in our model. 

An unexpected observation was that vehicle-treated *zak−/−* mice had a histopathology score comparable to ricin-intoxicated *zak+/+* mice, suggesting that *zak*-knockout mice have baseline epithelial abnormalities. We speculate that ZAK deficiency may result in changes to the host microbiota or a perturbed innate immune response to host microbiota. Another possibility resulting in abnormal histopathology is that ZAK deficiency results in defects in duodenal epithelial growth, structure, and/or barrier function. This may be possible, as ZAK signaling has been shown to influence cell proliferation and migration [28,35,36]. We also speculate that during ricin intoxication of *zak−/−* mice, the baseline abnormalities seen in vehicle-treated *zak−/−* mice may be diminished in the short term as signaling resources are redirected. This effect combined with the lack of a sufficient RSR may result in the low histopathology score we obtained for ricin-intoxicated *zak−/−* mice at 22 h. In this scenario, cellular damage due to ricin-mediated protein synthesis inhibition would be more pronounced in *zak−/−* mice at latter time points approaching 36 to 48 h when mortality has been predicted to occur in this model [23]. Finally, it could be that the specific cellular and immunological attributes of the epithelial abnormalities are different between ricin treatment in *zak+/+* mice and baseline duodenal abnormalities in *zak−/−* mice, with ricin-mediated pathology being due to inflammation and apoptosis, both of which are positively influenced by the RSR in vitro [15,29]. However, we are unclear as to the cause of the baseline abnormalities in *zak−/−* animals. It is possible that the differences are not discernable by our histopathology scoring methodology, and future studies will include looking specifically for intraepithelial immune cell infiltration and epithelial apoptosis. Efforts are ongoing to define the etiology of the baseline epithelial abnormalities observed in *zak−/−* duodenal tissues. 

There are currently no therapeutics that specifically treat ricin intoxication. We assessed only BMDMs and gross duodenal pathology, so it is not clear that all intoxicated cells in *zak−/−* mice are unable to mount an RSR. However, macrophage-induced inflammatory signaling is integral to ricin-associated pulmonary damage [24] such that we would expect pulmonary ricin intoxication to result in less morbidity and mortality in *zak−/−* mice. Future studies will be aimed at characterizing macrophage and neutrophil responses in vivo using our novel *zak−/−* murine strain together with oral and inhalational models of ricin intoxication. 

## 4. Conclusions 

In conclusions, our data describe a *zak−/−* mouse with a distinct RSR-associated phenotype, and support its use as a novel model for future studies of ZAK signaling. In addition to ricin, several other agents induce a ribotoxic stress response including Shiga toxins, doxorubicin, anisomycin, and the trichothecene toxins such as deoxynivalenol [15,37,38,39]. Therefore, our murine strain may prove valuable to studying the disease mechanisms associated with these agents. In addition to the ribotoxic stress response, ZAK has been associated with cell cycle arrest, various malignancies, cellular migration, and heart failure [26,27,28,36,40,41,42,43]. Therefore our *zak*-knockout mouse is a novel tool, which may benefit studies of these phenomena as well.

## 5. Materials and Methods 

### 5.1. Mice 

*zak+/−* mice were developed at Texas Institute for Genomic Medicine (TIGM) (College Station, TX, USA). A murine embryonic stem cell line was generated by TIGM in a 129S5/SvEvBrd genetic background with a gene trap insertion in *zak*. Resulting *zak+/−* mice were delivered to Tufts in a 50% 129S5/SvEvBrd and 50% C57BL/6 genetic background. Wild type *zak+/+* C57BL/6 mice for experiments and backcrossing were obtained from the Jackson Laboratory (Bar Harbor, ME, USA). “129/B6”or “B6” are used throughout the manuscript and supplementary figures to designate strains that are of a 129S5/SvEvBrd and C57BL/6 mixed (129/B6) genetic background or of a wild type C57BL/6 (B6) genetic background respectively. This is done to discriminate between data generated from non-congenic animals in the supplementary data versus that generated from wild-type *zak+/+* B6 mice and backcrossed congenic *zak-/-* B6 mice, as used for all data in Figure 1 and Figure 2. Prior to commencement of the work, all live animal procedures were pre-approved by Tufts University institutional animal care and use committee (Protocol # B2010-111, approved 17 August 2010). Oral ricin intoxication was performed according to Yoder et al. [23]. Briefly, mice at 8–11 weeks were fasted for 1 h prior to gavage with water provided ad libitum. Mice were dosed with ricin (10 mg/kg body weight), which was diluted in phosphate buffered saline (PBS), or PBS (vehicle) by oral gavage. At approximately 22 h post gavage, mice were sacrificed by CO_2_ asphyxiation. Duodenal tissue (i.e., 1 cm most proximal portion of the small intestine was placed in Bouin’s fixative for at least 24 h, and then tissue was transferred to 70% ethanol. Hematoxylin and eosin (H&E) stained cross-sections of the duodenum were blindly scored by one of us (N.J.M.) for ricin intoxication according to a 12-point histologic grading system, which was based on the severity and extent of alterations in villus shape (destruction of tips, width and height), lamina propria edema (venules with RBCs), interepithelial swelling, and the presence of cellular infiltrate (e.g., PMNs) in the intestinal lumen, as described by others [23,44]. Tissue section samples were coded prior to blinded scoring. Each slide had multiple sections/slide and typically one slide per animal was scored. 

### 5.2. Harvesting of Bone Marrow–Derived Macrophages (BMDMs)

Mice between nine and 12 weeks old were asphyxiated with CO_2_ followed by cervical dislocation. Femurs were cut at the ends, and as previously described (17), marrow was flushed with BM-extraction medium consisting of α-minimal essential medium (Cellgro, Corning, Manassas, VA, USA) supplemented with 10% FBS (Gibco, ThermoFisher Scientific, Waltham, MA, USA), 50 μg/mL gentamicin (Life Technologies, Beverly, MA, USA), 100 ng/mL recombinant mouse colony-stimulating factor 1 (R&D Systems, Minneapolis, MN, USA), and 1X Fungizone (2.5 μg/mL amphotericin B and 2.05 μg/mL sodium deoxycholate) (Gibco, ThermoFisher Scientific, Waltham, MA, USA). Cells were cultured on 100 mm × 15 mm polystyrene Petri dishes (Fisherbrand, cat. #FB0875713, ThermoFisher Scientific, Waltham, MA, USA) for 72–96 h. The cells were passaged onto new Petri dishes and cultured in BM-culture medium (consisting of α-minimal essential medium supplemented with 10% FBS, 50 μg/mL gentamicin, and 100 ng/mL recombinant mouse colony-stimulating factor 1 and expanded as needed. For Figure 1 panel A, growth media was replaced with serum-free BM-culture medium 30 min prior to adding ricin, however, similar results had been obtained using serum-containing medium (Figure S3, Panel A).

### 5.3. Ricin

Unconjugated Ricinus Communis Agglutinin II (RCA II, RCA60, ricin) was purchased from Vector Labs (Burlingame, CA, USA) catalog number L-1090. Ricin was dialyzed against PBS to remove azide. 

### 5.4. Preparation of Whole Cell Extracts, MAPK Immunoprecipitation Assays, and Western Blotting 

Following experimental treatments, culture dishes were transferred to ice. Media was removed and cells were scraped off the culture dishes in ice-cold Dulbecco’s PBS (without Ca^2+^ or Mg^2+^) (Gibco, ThermoFisher Scientific, Waltham, MA, USA) containing leupeptin 10 μg/mL, 1 mM PMSF, and 0.5 mM DTT. Cells were centrifuged at 4 °C for 2 min at 10,000 rpm in a microfuge. The supernatant was removed, and pelleted cells were resuspended in lysis buffer consisting of 0.1% Triton X-100, 25 mM HEPES (pH 7.5) (Gibco, ThermoFisher Scientific, Waltham, MA, USA), 300 mM NaCl, 1.5 mM MgCl_2_, 200 μM EDTA (pH 8.0) (Ambion, ThermoFisher Scientific, Waltham, MA, USA), 0.1 mM Na_3_VO_4_, 20 mM β-glycerol phosphate, 10 μg/mL leupeptin, 1 mM PMSF, and 0.5 mM DTT. Unless noted otherwise, reagents used to generate whole cell extracts were purchased from Sigma-Aldrich, Inc. (St. Louis, MO, USA). The suspended cells were lysed by gently rocking at 4 °C for 30 min. Cellular debris was removed by centrifugation at 4 °C at >14,000 rpm in a microfuge, and supernatants were collected and stored at −80 °C. Protein concentrations of cell extracts were estimated using Bio-Rad (Hercules, CA, USA) protein assay reagent according to the manufacturer’s instructions.

Based on protein assay results, lysates with approximately equal amounts of protein were loaded on an SDS-PAGE gel followed by transfer to PVDF membrane. Antibodies used for Western blotting: Anti-phosphorylated-JNK1/2 (cat. #9255s) and Phosphorylated-p38 (cat. #9211s) were purchased from Cell Signaling Technology (Danvers, MA, USA), and anti-GAPDH antibody (cat. #SC25778) from Santa Cruz Biotechnology Inc. (Dallas, TX, USA).

### 5.5. Quantitative Real-Time PCR

Purification of RNA was performed using the Qiagen (Valencia, CA, USA) RNeasy Plus Mini Kit cat. #74134. qRT-PCR was performed on RNA using Power SYBR Green RNA-to-CT (ThermoFisher Scientific cat. #4389986, Waltham, MA, USA). All reactions were performed on a CFX Connect Real-Time System (Bio-Rad, Hercules, CA, USA). 5 ng of total cellular RNA was used for each reaction. Primers for *β-actin* (reference gene), *cxcl1* and *c-jun* were purchased from Qiagen, catalog numbers QT00095242, QT00115647, and QT00296541 respectively. The primers for measuring *zak* expression were purchased from Integrated DNA Technologies (Coralville, IA, USA) assay ID# Mm.PT.58.31046083. The values for relative copies (mRNA) are based on calculation of ∆∆CT normalized to that from untreated BMDMs from C57BL/6 mice. 

## Supplementary Materials

The following are available online at www.mdpi.com/2072-6651/8/9/259/s1., Figure S1: BMDMs from 129/B6 *zak−/−* mice fail to induce the RSR but activate JNKs and p38 in response to LPS. (**A**) Western blots for phosphorylated JNK1/2 and phosphorylated p38 performed following treatment of *zak+/+* (wt) B6 or *zak−/−* 129/B6–derived BMDMs with 10 ng/mL ricin for periods of time ranging from 0.5 to 4 h. (**B**) Western blots for phosphorylated JNK1/2 and p38 performed following treatment with 50 ng/mL LPS for periods of time ranging from 0.5 to 4 h. Band intensities were measured using ImageJ 1.46r software (National Insitutes of Health, Bethesda, MD, USA). Phosphorylated p38 and JNK1/2 band intensities were normalized to respective loading controls (i.e., total p38) and depicted in the graphs below the Western blots. Figure S2: BMDMs from noncongenic 129/B6 *zak−/−* mice have decreased induction of proinflammatory cytokines compared to those from *zak +/+* B6 (wt) mice following intoxication with the ribotoxic stressors ricin or deoxynivalenol (DON). (**A**) qRT-PCR for *il-1β*, *cxcl1*, and *tnf-α* following treatment with LPS or intoxication with ricin. Gene expression for all three cytokines is decreased in BMDMs from noncongenic *zak−/−* 129/B6 mice following ricin treatment but not LPS as compared to BMDMs from wt mice. (**B**) Following intoxication with the trichothecene toxin DON, relative expression of *il-1β*, *il-6*, *tnf-α*, *cxcl1*, and *ccl2* but not *il-1α* was lower in BMDMs from noncongenic *zak−/−* 129/B6 mice, compared to BMDMs from wt mice. Figure S3: BMDMs from congenic B6 *zak−/−* and B6 *zak+/+* (wt) mice demonstrate a perturbed or ablated RSR when treated with ricin in the presence or absence of serum. (**A**) Western blot for Phosopho-JNK1/2. (**B**) Western blot for Phospho-p38. Lysates for panels A and B were made from BMDMs intoxicated with ricin in BM culture medium. (**C**) Western blots for Phosopho-JNK1/2 and Phospho-p38. Lysates were made from BMDMs intoxicated with ricin in serum-free culture medium. GAPDH is used as a loading control in all blots.

## Abbreviations

The following abbreviations are used in this manuscript:

MAPK	Mitogen activated protein kinase
MAP3K	Mitogen activated protein 3 kinase
BMDMs	Bone Marrow Derived Macrophages
RSR	Ribotoxic Stress Response
qRT-PCR	quantitative real-time polymerase chain reaction
