# Supplementary material for: A Novel Zak Knockout Mouse with a Defective Ribotoxic Stress Response
Source: Toxins (Basel). 2016 Sep 2;8(9):259. doi: 10.3390/toxins8090259 (PMC5037485; doi:10.3390/toxins8090259)
Supplement: Supplementary file 1 [file toxins-08-00259-s001.pdf]

# Supplementary Materials: A Novel *Zak* Knockout Mouse with a Defective Ribotoxic Stress Response

**Dakshina M. Jandhyala, John Wong, Nicholas J. Mantis, Bruce E. Magun, John M. Leong and Cheleste M. Thorpe**

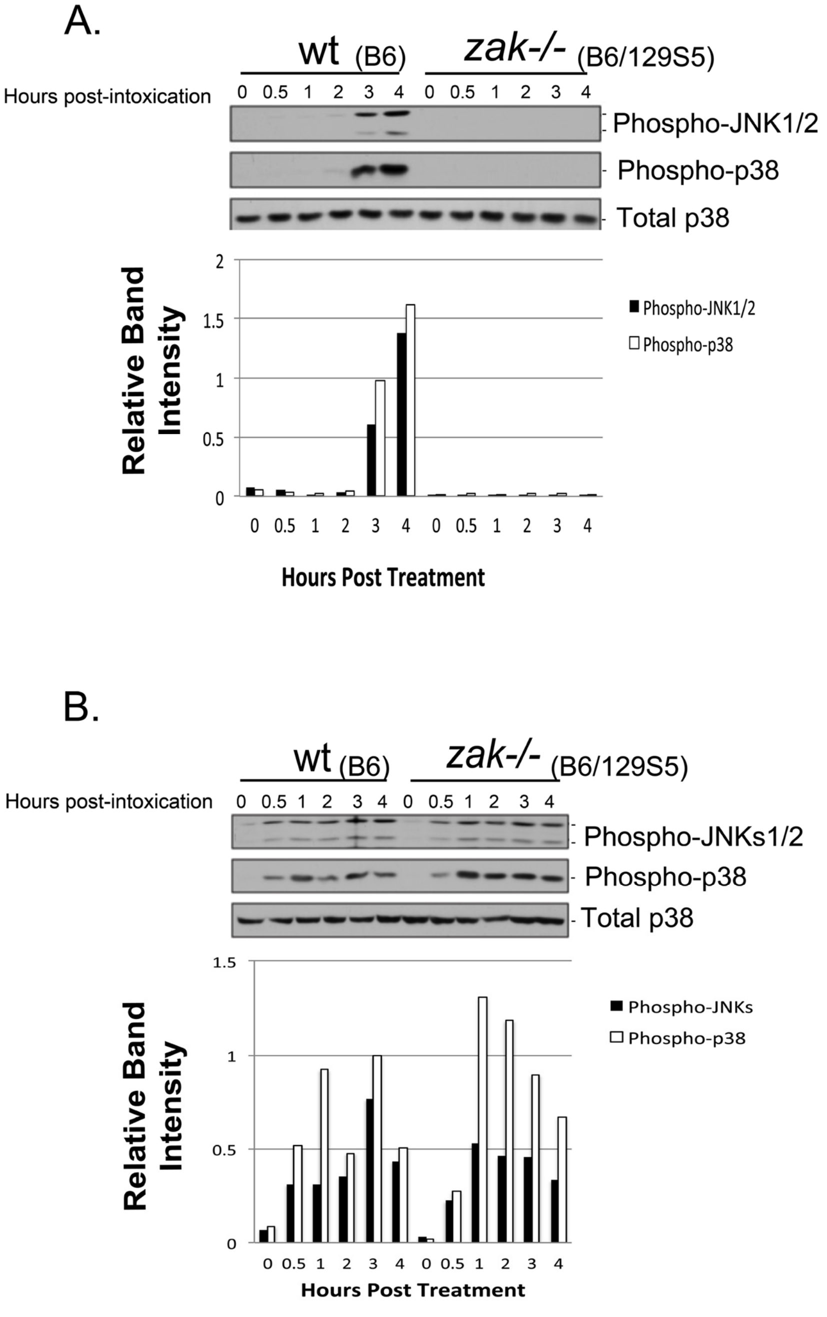


**Figure S1.** BMDMs from 129/B6 *zak*−/− mice fail to induce the RSR but activate JNKs and p38 in response to LPS. (**A**) Western blots for phosphorylated JNK1/2 and phosphorylated p38 performed following treatment of *zak*+/+ (wt) B6 or *zak*−/− 129/B6–derived BMDMs with 10 ng/mL ricin for periods of time ranging from 0.5 to 4 h. (**B**) Western blots for phosphorylated JNK1/2 and p38 performed following treatment with 50 ng/mL LPS for periods of time ranging from 0.5 to 4 h. Band intensities were measured using ImageJ 1.46r software (National Insitutes of Health, Bethesda, MD, USA). Phosphorylated p38 and JNK1/2 band intensities were normalized to respective loading controls (i.e. total p38) and depicted in the graphs below the Western blots.

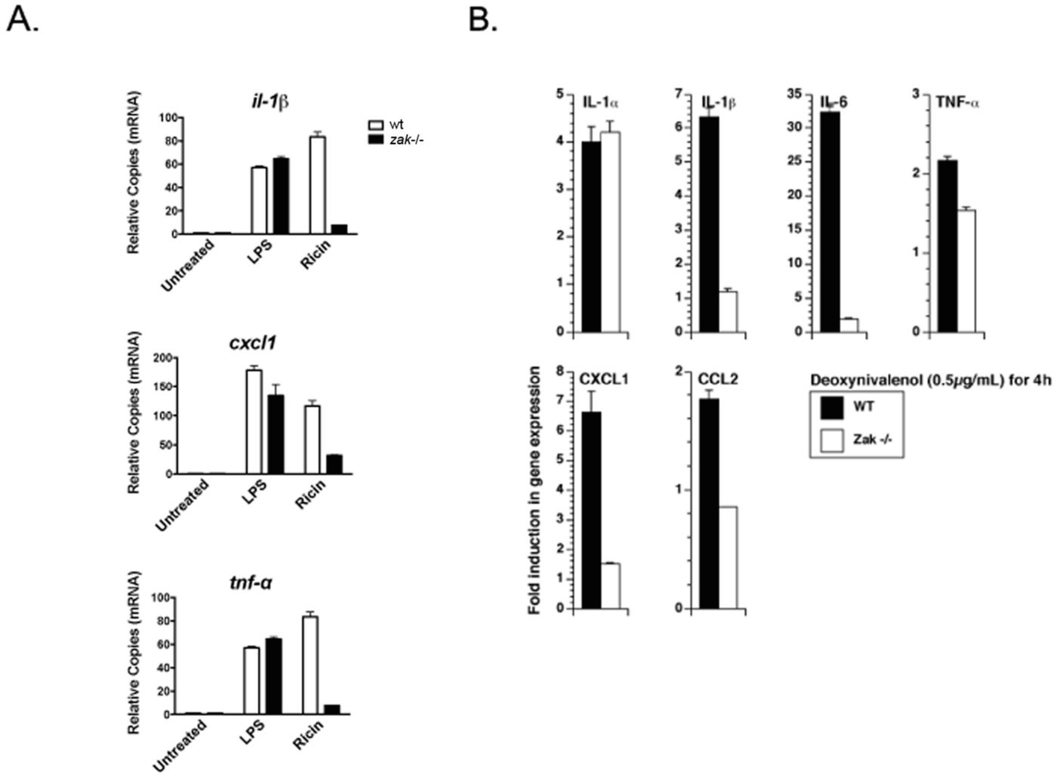


**Figure S2.** BMDMs from noncongenic 129/B6 *zak*−/− mice have decreased induction of proinflammatory cytokines compared to those from *zak* +/+ B6 (wt) mice following intoxication with the ribotoxic stressors ricin or deoxynivalenol (DON). (**A**) qRT-PCR for *il-1β*, *cxcl1*, and *tnf-α* following treatment with LPS or intoxication with ricin. Gene expression for all three cytokines is decreased in BMDMs from noncongenic *zak*−/− 129/B6 mice following ricin treatment but not LPS as compared to BMDMs from wt mice. (**B**) Following intoxication with the trichothecene toxin DON, relative expression of *il-1β*, *il-6*, *tnf-α*, *cxcl1*, and *ccl2* but not *il-1α* was lower in BMDMs from noncongenic *zak*−/− 129/B6 mice, compared to BMDMs from wt mice.

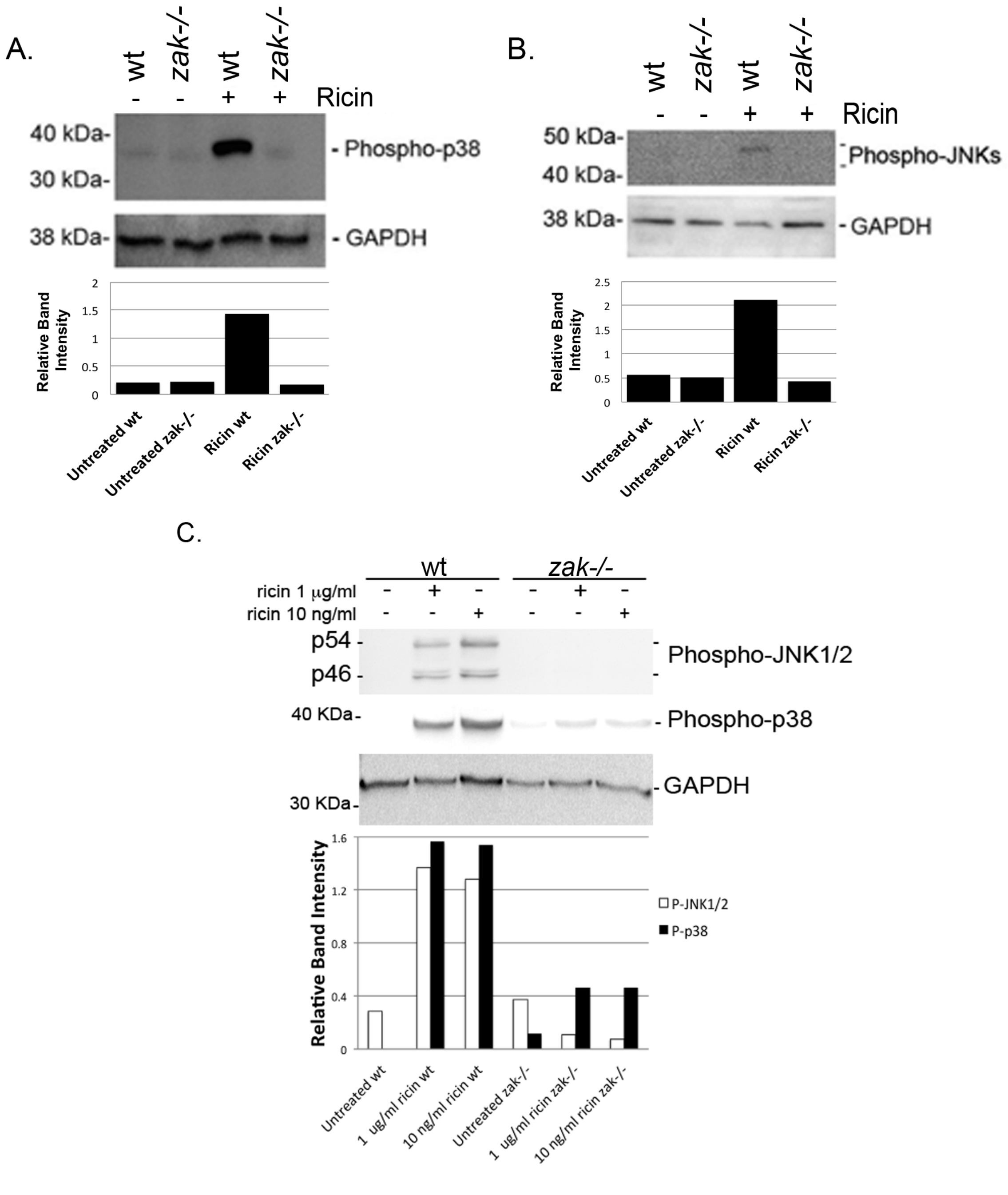


**Figure S3.** BMDMs from congenic B6 *zak*−/− and B6 *zak*+/+ (wt) mice demonstrate a perturbed or ablated RSR when treated with ricin in the presence or absence of serum. (**A**) Western blot for Phosopho-JNK1/2. (**B**) Western blot for Phospho-p38. Lysates for panels A and B were made from BMDMs intoxicated with ricin in BM culture medium. (**C**) Western blots for Phosopho-JNK1/2 and Phospho-p38. Lysates were made from BMDMs intoxicated with ricin in serum-free culture medium. GAPDH is used as a loading control in all blots.
